# Immigrants’ Enforcement Experiences and Concern about Accessing Public Benefits or Services

**DOI:** 10.1007/s10903-023-01460-x

**Published:** 2023-03-01

**Authors:** Lei Chen, Maria-Elena De Trinidad Young, Michael A. Rodriguez, Kathryn Kietzman

**Affiliations:** 1 Department of Social Welfare, Luskin School of Public Affairs and Center for Health Policy Research, University of California Los Angeles, Los Angeles, CA USA; 2 Department of Public Health, School of Social Sciences, Humanities and Arts, University of California Merced, 5200 Lake Road, Merced, CA 95343 USA; 3 Department of Family Medicine, David Geffen School of Medicine, University of California Los Angeles, Los Angeles, CA USA; 4 Center for Health Policy Research, University of California Los Angeles, Los Angeles, CA USA

**Keywords:** Law enforcement, Public charge, Latinx and Asian immigrants, Immigration Policies, Healthcare access

## Abstract

Although exclusionary immigration policies are associated with fear of deportation and avoidance of public benefits, relationships between immigration enforcement policy and public charge policies are largely unknown. Using a California population-based survey of 1103 Asian and Latinx immigrants in 2018, we tested the relationship between immigrants’ experiences with law enforcement and their concern about public charge. Direct encounters with various forms of law enforcement, including being asked to show proof of citizenship by law enforcement, staying inside to avoid police or immigration officials, and having known someone who had been deported, were associated with immigrants’ avoidance of public benefits due to public charge concerns. Latinx immigrants were more likely to be concerns about public charge than Asians. Intersections among immigration policies deserve further consideration. There is a need to provide accurate and reliable information to immigrant communities about public benefits and advocate for inclusive immigration policies.

## Background

The United States has a long history of enacting restrictive, exclusionary immigration policies [1]. In the last several decades, federal policy has both curtailed noncitizens’ eligibility for public benefits and expanded immigration enforcement powers [2]. For example, The Personal Responsibility and Work Opportunity Reconciliation Act (PRWORA), signed in 1996, restricted some lawful immigrants’ access to certain public programs and denied undocumented immigrants’ access to most federally funded programs. At the same time, in 1996 the *Illegal Immigration Reform and Immigrant Responsibility Act (IIRIRA)* was also signed into law, to fund more immigration enforcement activities and expand the grounds for deportation, placing immigrants, especially undocumented immigrants, at greater risk for arrest, detention, and deportation [2–4]. These policies have been associated with a “chilling effect” in which immigrants’ fears and concerns about the negative consequences of immigration policy result in avoidance of healthcare services and other resources [3, 4]. However, few empirical studies have examined the intersections of these exclusionary policies’ impact on immigrants’ access to healthcare and other services. In this study, we sought to understand the associations between the impact of exclusionary enforcement policies and exclusionary safety net policies, with a focus on concerns about the public charge rule. We examined Latinx and Asian immigrants’ experiences of enforcement policies and their association with concerns about accessing public benefits or services.

It is believed that “chilling effects” directly result from the public charge rule. The public charge rule refers to criteria by which applications for lawful permanent residence (LPR, often referred to as a green card) or temporary visas are deemed “likely to become a public charge” and can be denied [5]. It was first introduced into federal legislation as part of the Immigration Act of 1882 [6]. Public charge was the most common reason used to refuse noncitizens admission at U.S. ports of entry in the late 19th and early twentieth centuries [6]. The Trump administration expanded the reach of public charge to other benefits in 2019 (e.g., receiving food assistance, Medicaid, housing assistance). This public charge rule took effect in February 2020 until President Biden formally rescinded it on March 11, 2021. The recent introduction and enactment of changes to the public charge rule were associated with reductions in immigrants’ access to services, raising concerns that immigrants and their families, including legal citizens, would be at greater risk for adverse health outcomes [5–10].

Immigration enforcement policies may be associated with or exacerbate barriers to immigrants’ use of public benefits [11, 12]. Immigration enforcement policies have increased surveillance and policing by both immigration authorities and local law enforcement and have increased deportation of noncitizens by immigration authorities [13–15]. Immigration enforcement policies elicit stress and fear among immigrants, which in turn affect immigrants’ behaviors and experiences [3]. These behaviors and experiences are often interrelated; beyond avoiding public benefits, they may include avoiding police or immigration officials, being deported, and being racially profiled by law enforcement [11, 12, 16–18]. Findings in previous studies suggest that direct experiences with specific types of immigrant enforcement (e.g., the risk of being deported, heightened law enforcement activity, ICE enforcement of deportation policies), as well as perceptions regarding enforcement (e.g., feeling unsafe living in the U.S.) may deter immigrants from seeking public services and healthcare [4, 14, 15, 19–23]. Previous qualitative research has found that barriers to immigrants’ access to social services and healthcare include immigrant policing, fear of deportation, interaction with law enforcement, and deterring the settlement of unauthorized immigrants [16, 24–26]. However, these studies focus on single instances of immigration enforcement or immigrants’ perceptions; no study has comprehensively examined various exclusion experiences of law enforcement and their association with immigrants’ concerns about public charge. Furthermore, we are unaware of population-based studies that have investigated this association among Latinx and Asian immigrants.

In addition, most studies related to law enforcement have focused on Latinx immigrants and there is a notable lack of studies that include Asian immigrants [21–24, 27–29]. Asian immigrants are the fastest-growing and second-largest foreign-born population (31%) in the United States and in California (39%) [30, 31]. California is one of a growing number of “majority-minority” states, and has a significant representation of immigrants, particularly Latinx and Asian immigrants.

To fill these gaps, this paper uses statewide population-level data to assess the extent to which various law enforcement experiences were associated with immigrants’ concerns about public charge, as measured by Latinx and Asian immigrants’ self-report of avoidance of public benefit use due to concerns about their legal status in California. We also considered immigrants’ social locations (e.g., race/ethnicity, citizenship/legal status, employment, education, poverty levels, years in the U.S., and languages), that have been previously associated with immigrants’ experiences of fear and chronic stress [3, 15, 19, 25–27].

## Methods

### Datasets

This paper used data from the Research on Immigrant Health and State Policy (RIGHTS) Study, which sought to understand how California’s immigrant policy environment influenced immigrants’ access to healthcare. The RIGHTS survey was a follow-up to the 2018 California Health Interview Survey (CHIS), the largest state-level population health survey in the U.S, collecting data on access to care, socio-demographic, immigration, and health status characteristics. The random-digit-dial phone survey of the RIGHTS study was administered to a representative, follow-up sample of Latinx and Asian immigrants throughout California who participated in CHIS in 2018 (*N* = 1,103). RIGHTS participants completed the survey by phone approximately 1–3 months after completing CHIS. The 2018 RIGHTS survey data was collected from September 2018 to February 2019. This paper used merged 2018 RIGHTS and CHIS survey data for analyses. The study was reviewed and approved by the Institutional Review Board at the [REDACTED].

### Measures

The dependent variable to measure immigrants’ concerns about public charge came from the RIGHTS survey. Respondents were asked “Was there ever a time when you decided not to apply for one or more government services, such as Medi-Cal, food stamps, or housing subsidies, because you were worried it would disqualify you, or a family member, from obtaining a green card or becoming a U.S. citizen?” The answer to this question was no = 0 or yes = 1.

We examined 7 independent variables that each measured a distinct type of experience of law enforcement (Table 1). The measures came from 7 items in the RIGHTS survey in which respondents were asked to report (no = 0 or yes = 1) if they had ever had each experience. We used each survey response as a binary variable.

**Table 1:** Measurement of immigrants’ experiences with law enforcement

Measures	RIGHTS survey questions
Staying inside to avoid police or immigration officials	Has there ever been a time when you decided not to leave your house or stayed away from certain areas to avoid the police or immigration authorities?
Having seen immigration officials in one’s own neighborhood	In the past 12 months, have you seen immigration authorities in your neighborhood?
Having been watched by law enforcement	Have you ever been watched by a law enforcement officer on the street or a public place?
Having been racially profiled by law enforcement	Have you ever been stopped for no good reason by law enforcement?
Having been asked to show proof of citizenship by law enforcement	Have you ever been asked to show proof of your citizenship or legal status by a police officer or other law enforcement authority?
Having been deported	Have you ever been deported?
Having known someone who had been deported	Do you know anyone personally who has ever been deported?

Immigrants’ social locations refer to their demographic and socioeconomic status, including race/ethnicity, citizenship/legal status, age, gender, cohabitation, education, employment, and poverty level. All of these variables came from the CHIS dataset. Among the demographic variables, the variable of race/ethnicity was reclassified into two categories based on respondents’ self-reported country of origin: Latinx (born in any country of Latin America or the Caribbean) = 1 and Asian (born in any country of Asia, except for the Middle East) = 0. The variable of citizenship/legal status included immigrants’ status when completing the survey as naturalized citizen = 0; non-citizen with green card = 1; non-citizen without green card = 2. We included variables for self-reported age (continuous), self-reported gender (male = 0; female = 1), and cohabitation (Widowed/Separated/Divorced and never married = 0; Married or living with partner = 1). For socioeconomic status, we included education (below high school education = 0; high school education or above = 1), employment (in labor market = 0; not in labor market = 1; unemployed = 2), below 200% federal poverty level (no = 0; yes = 1), years in the U.S. (less than 5 years = 0; 5 years and above = 1), and interview language (non-English = 0; English = 1).

### Analysis

Given the binary nature of the outcome variable, we constructed two phases of logistic regression models. In the first phase, we tested a single logistic regression model to examine the association between respondents’ social locations and their concerns about public charge. In the second phase, we tested seven individual logistic regression models to examine the association between each individual experience of law enforcement and respondents’ concerns about public charge, controlling for their social locations. All analyses were weighted to account for sampling design and to produce population-based estimates.

### Sensitivity Analysis

Because our measure of citizenship/legal status could not distinguish if a respondent was currently undocumented, we conducted sensitivity analyses using a variable from the RIGHTS survey that assessed if respondents had ever been undocumented. In the RIGHTS survey, respondents reported if there was ever a time they did not have a valid visa or other document which permitted them to stay in the United States (no = 0; yes = 1). Following the second phase, for each logistic model we replaced the citizenship/legal status variable with this ever-undocumented variable, maintaining all other variables. The sensitivity analysis showed that the significance pattern for individual law enforcement experience was consistent with each original model. Because the ever-undocumented variable did not significantly change the results, we proceeded with the analyses without further use of this variable.

## Results

### Descriptive Analysis

Nearly one-quarter (23.5%) of respondents reported that they had not applied for public benefits or services due to concerns about public charge (Table 2). Regarding the individual experiences of law enforcement: 31.0% of respondents reported having known someone who had been deported, which was the most common experience; 15.2% of respondents said that there was a time when they decided not to leave their house or stayed away from certain areas to avoid the police or immigration authorities; 13.3% of respondents had been racially profiled; 11.8% had been watched by a law enforcement officer on the street or a public place; 9.6% of respondents had seen immigration officials in their neighborhood in the last year; 6.4% of respondents had been asked to show proof of citizenship or legal status by a police officer or other law enforcement authority; and 3.1% of respondents had been deported, which was the least common experience. On average, respondents reported having one type of law enforcement experience.

**Table 2:** Descriptive analysis of dependent, independent and social location variables (n = 1103)

Indicator		Category	Percent
Concerns about public charge		Yes	23.5
Experiences of law enforcement	Staying inside to avoid police or immigration officials	Yes	15.2
	Having seen immigration officials in one’s own neighborhood	Yes	9.6
	Having been watched by law enforcement	Yes	11.8
	Having been racially profiled by law enforcement	Yes	13.3
	Having been asked to show proof of citizenship by law enforcement	Yes	6.4
	Having been deported	Yes	3.1
	Having known someone who had been deported	Yes	31.0
	Number of law enforcement experiences		0.9 (Mean)
Social locations	Race/ethnicity	Asian	41.4
		Latinx	58.6
	Citizenship/Legal status	Naturalized citizen	53.6
		Non-citizen with green card	25.6
		Non-citizen without green card	20.8
	Self-reported age	49.0 (mean)	
	Self-reported gender	Male	46.4
		Female	53.6
	Cohabitation	Yes	67.3
		No	32.8
	Education	High school education or above	65.3
		Below high school education	34.7
	Employment	In labor market	58.6
		Not in labor market	37.3
		Unemployment	4.1
	FPL < 200%	Yes	53.6
		No	46.5
	Years in the U.S	Less than 5 years	5.7
		5 years and above	94.3
	Interview language	Non-English	52.6
		English	47.4

The distributions of the social location variables show that more than half of the respondents (58.6%) were born in Latin America and 41.4% were born in Asia. More than half of the respondents (53.6%) were naturalized citizens, 25.6% were non-citizens with green cards, and 20.8% were non-citizens without green cards. The average age of the respondents was 49 years. Slightly more than half (53.6%) self-reported as female. Two-thirds of the respondents were living with a partner. Two-thirds had a high school education or above. More than half of the respondents (58.6%) were employed, while 4.1% were unemployed and one-third were not in the labor market (37.3%). More than half (53.6%) of respondents were living below the 200% federal poverty level. Most of the respondents (94.3%) lived in the U.S. 5 years or more. More than half (52.6%) of respondents responded to the RIGHTS survey in a language other than English.

### Associations Between Social Locations and Concerns About Public Charge

Table 3 shows the model testing the associations between social location variables and immigrants’ concerns about public charge. Latinx respondents had three times the odds of having not applied for public benefits or services due to concerns about public charge compared to Asian respondents, net all other variables (OR = 3.0, *p* < 0.01). Citizenship/legal status was not significantly associated with respondents’ public charge concerns, net all other variables. Respondents who cohabitated with a partner had almost two times the odds of having been concerned about public charge than those who did not cohabitate, net all other variables (OR = 1.9, *p* < 0.05). Compared with respondents in the labor market, those who were not in the labor market had significantly lower odds of concerns about public charge, net all other variables (OR = 0.4, *p* < 0.01). The other social locations, such as age, gender, education, poverty level, years in the U.S., and interview language were not significantly associated with respondents’ concerns about public charge.

**Table 3:** Logistic regression between immigrants’ social locations and concerns about public charge (n = 1103)

	Odds ratio	95% Confidence interval	
Race/ethnicity			
Asian (ref)			
Latinx	3.0**	1.5—6.0	
Citizenship/Legal status			
Naturalized citizen (ref)			
non-citizen with green card	1.4	0.8—2.7	
non-citizen without green card	1.3	0.7—2.9	
Age	1.0	1.0—1.0	
Gender			
Male (ref)			
Female	1.4	0.8—2.3	
Cohabitation			
No cohabitation (ref)			
Cohabitation	1.9*	1.1—3.3	
Education			
Below high school education (ref)			
High school or above	1.1	0.6 – 2.1	
Employment			
In labor market (ref)			
Not in labor market	0.4**	0.2—0.7	
Unemployment	0.6	0.2—1.8	
Poverty level			
200% federal poverty level or above (ref)			
Below 200% federal poverty level	1.4	0.8—2.4	
Years in the U.S			
Less than 5 years (ref)			
5 years and above	2.0	0.8—5.4	
Interview language			
Non-English (ref)			
English	0.7	0.3, 1.3	
_cons	0.1	0.0—0.4	

*p < .01; **p < .05

### Associations Between Individual Enforcement Experience and Concerns About Public Charge

Table 4 shows the seven models testing the associations between each individual enforcement experience and concerns about public charge, net social location variables. Three law enforcement experiences were each significantly related to immigrants’ concerns about public charge: Having been asked to show proof of citizenship was associated with seven times the odds of having been concerned about public charge (OR = 7.2, *p* < 0.05); having ever stayed inside to avoid police or immigration officials had almost five times the odds of having been concerned about public charge than those who did not have this experience (OR = 5.1, *p* < 0.05); and knowing someone who was deported had three times the odds of having been concerned about public charge than those who did not have this experience (OR = 3.2, *p* < 0.05).

**Table 4:** Seven logistic regressions between immigrants’ individual enforcement experiences and concerns about public charge (n = 1103)

Law enforcement model^a^	Odds Ratio	95% Confidence Interval
Staying inside to avoid police or immigration officials	5.1**	2.6—9.9
Having seen immigration officials in one’s own neighborhood	1.1	0.6—2.2
Having been watched by law enforcement	2.0	1.0—4.2
Having been racially profiled by law enforcement	1.7	0.8—3.5
Having been asked to show proof of citizenship by law enforcement	7.2**	3.4—15.1
Having been deported	1.5	0.5—4.7
Having known someone who had been deported	3.2**	1.8—5.6

All the models controlled for race/ethnicity, citizenship/legal status, age, gender, cohabitation, education, employment, poverty level, years in the U.S., and interview language

**p < .05

Regarding social location variables (not shown), race/ethnicity remained significant in all models, except for the model for knowing someone who was deported. Cohabitation and employment remained significant in all seven models. The other variables (citizenship, age, education, poverty level, years in the U.S.) were not significant in any of the models.

## Discussion

In this study, we used population-based data on Latinx and Asian immigrants to examine the associations between their direct experiences with law enforcement and their concerns about accessing public benefits or services due to the public charge rule. Our findings indicate that three law enforcement experiences: being asked to show proof of citizenship by law enforcement, staying inside to avoid police or immigration officials, and having known someone who had been deported, were associated with immigrants choosing to avoid using public benefits or services due to their concerns about public charge. These enforcement experiences may be particular sources of stress and distrust for immigrants, creating uncertainty about their ability to access public benefits and services. For example, compared with other types of law enforcement experiences, being asked to show proof of citizenship by law enforcement officials is a direct and face-to-face interaction that may heighten risk of apprehension and deportation for undocumented immigrants and other noncitizens. This type of encounter may cause severe stress, fear, and anxiety for immigrants, further reducing their motivation to seek healthcare or other public services. The significant association found between staying inside to avoid police or immigration officials and concerns about public charge contributes to evidence that immigrants avoid public and other institutions when they are threats or risk of enforcement [32]. For example, evidence suggests that when enforcement is present in communities or during periods of ramped up exclusionary immigration policy, some immigrant families have avoided routine activities, such as interacting with teachers or school officials, healthcare providers, or the police [32]. Our finding that knowing someone who is deported is associated with concerns about public charge is consistent with mounting evidence that connections to an individual who has been deported is associated with worse outcomes among immigrants. For example, research found that knowing a deportee and/or someone who was undocumented was associated with poor mental health of Latinx and Asian adults [27, 28]. Our findings build on these existing studies to suggest that these direct encounters with enforcement are associated with concerns about the consequences of using public benefits and likely exacerbate existing barriers to health care and other health promoting resources. Further, our findings provide some of the first data that includes both Latinx and Asian immigrants, providing evidence that immigration enforcement experiences are not rare for either group and that enforcement policies may be a barrier to public benefits for both groups.

This study also found that Latinx immigrants were more likely to be concerned about public charge than Asian immigrants. This is similar to previous studies that have found a link between enforcement and avoidance of public resources among Latinx immigrants [21]. The current study’s findings indicate that immigration enforcement policies may create direct and indirect externalities that negatively affect Latinx immigrants’ families and communities [27]. Our findings also suggest that Latinxs and Asians likely contend with distinct types of vulnerability to public charge. However, when we examined the experience of having known someone who had been deported, unlike the other law enforcement experiences, there was no difference in the likelihood of having concerns about public charge for Latinxs and Asians. This suggests that the influence of knowing a deportee on immigrants’ concerns about public charge is not limited to a specific group of immigrants, although a higher percentage of Latinx immigrants (47%) reported knowing someone who had been deported than Asian immigrants (8%). The externalities of immigration policy are likely experienced across Asian and Latino communities, albeit in different ways. Although citizenship has long been considered an important social location when examining immigrants’ access to healthcare, we did not find a significant association between citizenship/legal status and public charge concerns [20]. This may be due to how we measured the concern about public charge, which is an “ever” question, while the measure of citizenship/legal status used in our analyses reflected their current situations, which might have changed over time. However, in our sensitivity analyses, accounting for respondents’ past undocumented status did not change the results. Also, this measure does not distinguish whether immigrants are undocumented or not. Another possible explanation is that people may have concerns about public charge regardless of their immigration and citizenship status. We found that employed immigrants were more likely to have concerns about public charge than immigrants who were not in the labor market. This is consistent with findings that essential immigrant workers might avoid public services/benefits because of the recent public charge rule at the beginning of 2020 [10]. We also found that immigrants who were married or living with partners were more likely to have concerns about public charge than immigrants who were widowed, separated, divorced or never married. This finding may help explain why married immigrants were less likely to use public services or programs (e.g., WIC and Medicaid), which were found in previous studies [4, 19]. All these findings suggest that the public charge rule could have ripple effects across an entire family: immigrants’ concerns about public charge may impact their family members’ use of public services or programs [7, 9].

The “chilling effect” has been discussed by others, who have demonstrated that immigrants who are eligible for public benefits and services fail to use them because they fear being labeled a public charge [33]. Our study contributes to explanations of the “chilling effect” by examining immigrants’ various enforcement experiences and their association to concerns about public charge. Our results showed that these co-occurring experiences are likely linked and should be understood together. Our findings can inform policymakers, researchers, and advocates who are concerned with the intersectionality of immigration policies that are related distinct forms of immigration exclusion—from law enforcement to public charge—in the United States. It is important to examine the impact of exclusionary policies together and push back on the anti-immigrant climate promoted by past administrations and support more inclusive policies and social attitudes moving forward [13]. Although the Biden administration has abandoned the public charge rule, the chilling effect may continue, especially for immigrants who have ever had enforcement experiences. Significant efforts will be required to decriminalize immigrants and people of color by abolishing immigration enforcement and creating a path to citizenship for all [13]. Extra efforts are also needed to support immigrant communities by providing accurate and reliable information, to alleviate their ongoing confusion, fear, and reluctance to enroll in benefits for which they are eligible [34].

## Limitations

One limitation of this study is that all but one of the law enforcement experiences were asked about whether immigrants “ever” had these experiences. Only the “seen immigration officials in their neighborhood” measure asked whether immigrants had experienced this in the last 12 months. The outcome of immigrants’ concerns about public charge was also assessed with an “ever” timeframe. It is hard to determine temporal ordering of immigrants’ law enforcement experiences and their concerns about public charge. As a result, in this cross-sectional analysis, we cannot establish nor test the temporality and causality of these associations. Another limitation is that the study used country of origin to measure race/ethnicity instead of directly asking respondents about their race/ethnicity. It only looked at Latinx and Asian immigrants and does not include other immigrants such as those from the Middle East or Africa. The diversity within Latinx and Asian immigrant groups cannot be addressed in this study, even though these two groups of people are heterogeneous, with different cultural practices/norms, different immigration experiences, and varying levels of health status [15, 35]. Further studies may be needed to examine the interactions and possible changes over time of immigrants’ enforcement experiences and concerns about public charge, while also considering their citizenship/legal status’ changes over time.

## New Contribution to the Literature

Despite these limitations, this study is among the first to quantitatively examine the associations between immigrants’ experiences with law enforcement and avoiding public benefits and services due to their concerns about public charge using a population-based dataset. This study expands our current knowledge of the potential intersections between enforcement and public charge policies that may impact immigrants’ behaviors and experiences. It also contributes to the explanations of the “chilling effect” beyond the public charge rule. The findings of this study reaffirm the critical need to examine the impact of enforcement experiences and concerns about public charge among multiple groups of immigrants, such as Latinx and Asian immigrants’, the largest and fastest growing racial/ethnic groups in the United States.
